# Neuropsychiatry Teaching for Medical Students: A Narrative Review

**DOI:** 10.1192/bjo.2024.327

**Published:** 2024-08-01

**Authors:** Philip Spani-Orchin, Elliott Tsai-Goodman, Mohan Rathnaiah

**Affiliations:** University of Nottingham, Nottingham, United Kingdom

## Abstract

**Aims:**

Neuropsychiatry is a new and burgeoning field of medicine that combines neuroscientific principles with neurology and psychiatric medicine. Currently, there is little to none medical school literature and/or teaching in the subject. Re-integration of Neurology and Psychiatry disciplines has been recommended, especially in undergraduate and graduate medical training as well as in research. Neuropsychiatry disorders are considered one of the most important causes of disability by the World Health Organization. As a concept, Neuropsychiatry is still not clear on a global scale, from neurological examination to medical school teaching. There have already been active efforts to design and implement Neuropsychiatry training to post-graduate trainees worldwide, particularly in USA, Australia and UK. However, there seems to be no such endeavours towards teaching medical students the role of the brain in the manifestation of neurological as well as psychiatry symptoms. We set out to complete a targeted literature review looking for Neuropsychiatry teaching, if any, in medical schools worldwide.

**Methods:**

A systematic literature search of relevant key phrases was carried out in PubMed and Google Scholar databases. These phrases were searched between 29–31 January 2024 aimed to encompass the full scope of available teaching resources and materials across psychiatry and neurosciences worldwide. These searches included:

1. (((Neuropsychiatry) AND (Medical students)) AND (Medical school)) AND (Medical education)
2. (((Neuropsychiatry education) AND (training)) AND (medical students)) AND (Medical education)
3. (Neuroscience-in-psychiatry) AND (medical school)
4. ((Neuropsychiatry) AND (Medical education)) AND (Medical students)
5. Further reading was completed from the selected articles (six in total).

**Results:**

A total of 324 results were found from systematic literature search after leaving out the duplicates, of which only 6 articles were included as relevant to aim of our study. None of the articles described clear Neuropsychiatry teaching to the medical students.

**Conclusion:**

Our review highlighted a distinct lack of Neuropsychiatry learning outcomes within medical school curriculum. Neuroscientific principles and methodologies are incorporated in treatment of patients, rationalising clear differentiation between neurology or psychiatry, but the overall picture from both disciplines and utilisation towards diagnosing and managing the cluster of symptoms manifesting from aberrant brain processes is still unclear. In line with previous research around education measurement, we propose that fundamentals from both Neurology and Psychiatry need to be introduced as clinical neuroscience early in medical school and this can be further continued.

